# Fenofibrate Regulates Visceral Obesity and Nonalcoholic Steatohepatitis in Obese Female Ovariectomized C57BL/6J Mice

**DOI:** 10.3390/ijms22073675

**Published:** 2021-04-01

**Authors:** Yujin Shin, Mijeong Lee, Dongju Lee, Joonseong Jang, Soon Shik Shin, Michung Yoon

**Affiliations:** 1 Department of Biomedical Engineering, Mokwon University, Daejeon 35349, Korea; ujin2821@naver.com (Y.S.); 12663@naver.com (M.L.); dlehdwn100@naver.com (D.L.); jeseell21@naver.com (J.J.); 2 Department of Formula Sciences, College of Korean Medicine, Dongeui University, Busan 47340, Korea

**Keywords:** fenofibrate, hepatic steatosis, hepatic inflammation, ovariectomized mice, PPARα, visceral obesity

## Abstract

Fibrates, including fenofibrate, are a class of hypolipidemic drugs that activate peroxisome proliferator-activated receptor α (PPARα), which in-turn regulates the expression of lipid and lipoprotein metabolism genes. We investigated whether fenofibrate can reduce visceral obesity and nonalcoholic fatty liver disease via adipose tissue PPARα activation in female ovariectomized (OVX) C57BL/6J mice fed a high-fat diet (HFD), a mouse model of obese postmenopausal women. Fenofibrate reduced body weight gain (−38%, *p* < 0.05), visceral adipose tissue mass (−46%, *p* < 0.05), and visceral adipocyte size (−20%, *p* < 0.05) in HFD-fed obese OVX mice. In addition, plasma levels of alanine aminotransferase and aspartate aminotransferase, as well as free fatty acids, triglycerides, and total cholesterol, were decreased. Fenofibrate also inhibited hepatic lipid accumulation (−69%, *p* < 0.05) and infiltration of macrophages (−72%, *p* < 0.05), while concomitantly upregulating the expression of fatty acid β-oxidation genes targeted by PPARα and decreasing macrophage infiltration and mRNA expression of inflammatory factors in visceral adipose tissue. These results suggest that fenofibrate inhibits visceral obesity, as well as hepatic steatosis and inflammation, in part through visceral adipose tissue PPARα activation in obese female OVX mice.

## 1. Introduction

Fibrates act as nuclear peroxisome proliferator-activated receptor α (PPARα) ligands and modulate the expression of genes that are crucial for lipid and lipoprotein metabolism, thereby contributing to lipid homeostasis [1,2]. Fibrate-activated PPARα forms heterodimers with the retinoid X receptor and binds to PPAR response elements in promoter regions of target genes, which have functions in the plasma triglyceride hydrolysis, fatty acid uptake and binding, and fatty acid β–oxidation [3,4,5]. The activation of PPARα target genes promotes the oxidation of fatty acids, increases the breakdown of triglycerides, and reduces triglyceride synthesis and secretion.

Fibrates, including fenofibrate, modulate body weight in obese animal models by regulating fatty acid oxidation and reducing the circulating triglyceride levels [6,7,8]. Likewise, PPARα-null mice have abnormalities in triglyceride metabolism, becoming obese with age [9]. Because impaired lipid metabolism leads to obesity and related metabolic diseases, such as dyslipidemia, atherosclerosis, type 2 diabetes, and nonalcoholic fatty liver disease (NAFLD) [10,11,12], improved PPARα activity is required to prevent or treat this group of disorders.

NAFLD is the common cause of liver diseases that range from simple steatosis to nonalcoholic steatohepatitis (NASH), fibrosis, and irreversible cirrhosis. NAFLD is associated with obesity and the metabolic complications of over-nutrition, which usually accompanies visceral obesity [13,14,15]. Human and animal studies have suggested that NAFLD is correlated with visceral adipose tissue inflammation and elevated circulating inflammatory factors, such as inflammatory adipokines and lipids [16,17,18]. Thus, decreasing visceral adipose tissue could be important for treating and preventing NAFLD.

Postmenopausal women have more visceral fat than premenopausal women and are at greater risk for metabolic complications associated with obesity [19,20]. Animal studies have demonstrated that ovariectomized (OVX) animals exhibit increased food intake, body weight, and body fat mass [21,22]. We evaluated the role of fenofibrate in obesity and NAFLD in female OVX mice, a mouse model of postmenopausal women. We found that fenofibrate not only inhibits visceral obesity and dyslipidemia but also reduces hepatic steatosis and inflammation in high-fat diet (HFD)-fed obese OVX mice. This process was likely mediated through visceral adipose tissue PPARα activation. Therefore, fenofibrate may be an effective treatment for postmenopausal women with obesity, hyperlipidemia, and NAFLD.

## 2. Results

### 2.1. Effects of Fenofibrate on Visceral Obesity and Adipocyte hypertrophy in HFD-Fed OVX Mice

**Figure 1:**
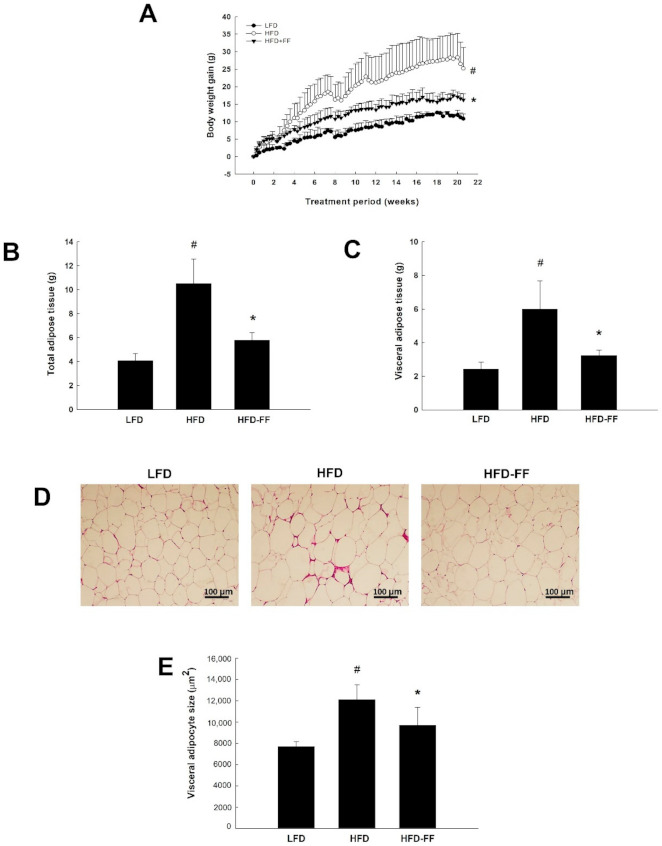
Effects of fenofibrate on body weight gain, adipose tissue mass, and visceral adipocyte size in HFD-fed female OVX mice. OVX C57BL/6J mice were fed an LFD, an HFD or an HFD supplemented with fenofibrate (HFD-FF, 0.05% *w*/*w*) for 21 weeks. (**A**) Body weight gains at the end of the treatment period are significantly different. (**B**) Total and (**C**) visceral adipose tissue mass. (**D**) Histology of visceral adipose tissue. Hematoxylin-eosin-stained visceral adipose tissue sections (original magnification ×100). (**E**) Visceral adipocyte size. All values are expressed as the mean ± SD (*n* = 8/group). # *p* < 0.05 compared with LFD. * *p* < 0.05 compared with HFD.

OVX mice that were fed an HFD supplemented with fenofibrate (HFD-FF mice; 16.80 ± 1.41 g) had lower body weight gains after 21 weeks of administration compared with HFD-fed mice (HFD mice; 27.10 ± 4.97 g) (Figure 1A). In obese mice, fenofibrate also significantly decreased total and visceral adipose tissue weights by 45% and 46%, respectively, (Figure 1B,C) and reduced the mean size of visceral adipocytes by 20% (Figure 1D,E). 

### 2.2. Effects of Fenofibrate on Plasma Levels of Liver Injury Markers and Lipids in HFD-Fed OVX Mice 

**Figure 2:**
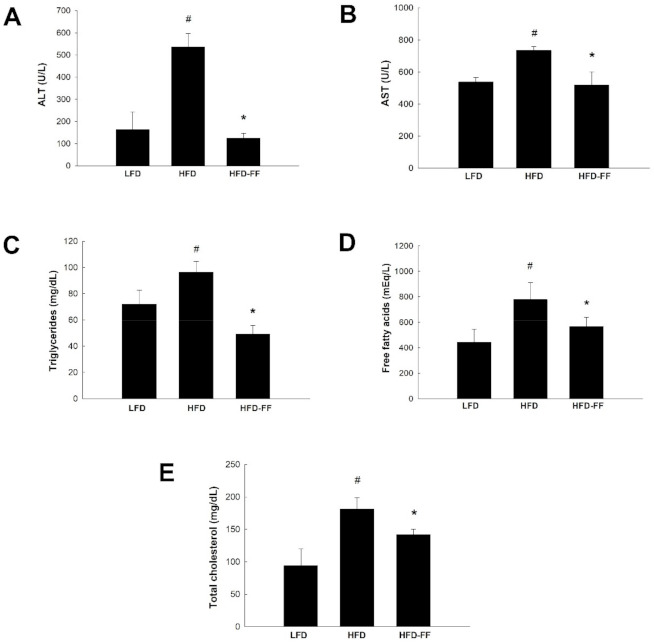
Effects of fenofibrate on circulating ALT, AST and lipid levels in HFD-fed female OVX mice. OVX C57BL/6J mice were fed an LFD, an HFD or an HFD supplemented with fenofibrate (HFD-FF, 0.05% *w*/*w*) for 21 weeks. Plasma levels of (**A**) ALT and (**B**) AST. Plasma levels of (**C**) triglycerides, (**D**) free fatty acids, and (**E**) total cholesterol. All values are expressed as the mean ± SD (*n* = 8/group). # *p* < 0.05 compared with LFD. * *p* < 0.05 compared with HFD.

Compared with low-fat diet (LFD)-fed mice (LFD mice), HFD mice had increased plasma concentrations of alanine aminotransferase (ALT) and aspartate aminotransferase (AST), and they were reduced by 76% and 29%, respectively, in HFD-FF mice compared with HFD mice (Figure 2A,B). Fenofibrate treatment also decreased plasma lipid levels, as indicated by reductions in triglycerides, free fatty acids, and total cholesterol by 49%, 27%, and 21%, respectively, in HFD mice compared to HFD mice (Figure 2C–E).

### 2.3. Effects of Fenofibrate on Obesity-Induced Hepatic Steatosis, Inflammation, and Hepatocyte Ballooning in HFD-fed OVX Mice

**Figure 3:**
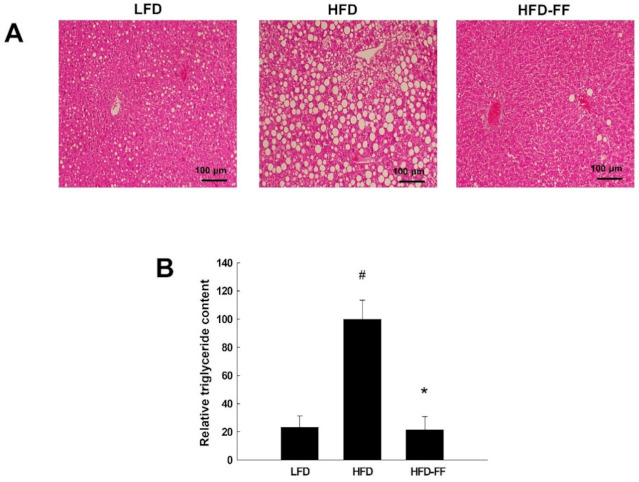
Effects of fenofibrate on hepatic lipid accumulation in HFD-fed female OVX mice. OVX C57BL/6J mice were fed an LFD, an HFD or an HFD supplemented with fenofibrate (HFD-FF, 0.05% *w/w*) for 21 weeks. (**A**) Hematoxylin-eosin-stained sections of liver tissues (original magnification ×100). (**B**) Relative triglyceride content. All values are expressed as the mean ± SD (*n* = 8/group). # *p* < 0.05 compared with LFD. * *p* < 0.05 compared with HFD.

**Figure 4:**
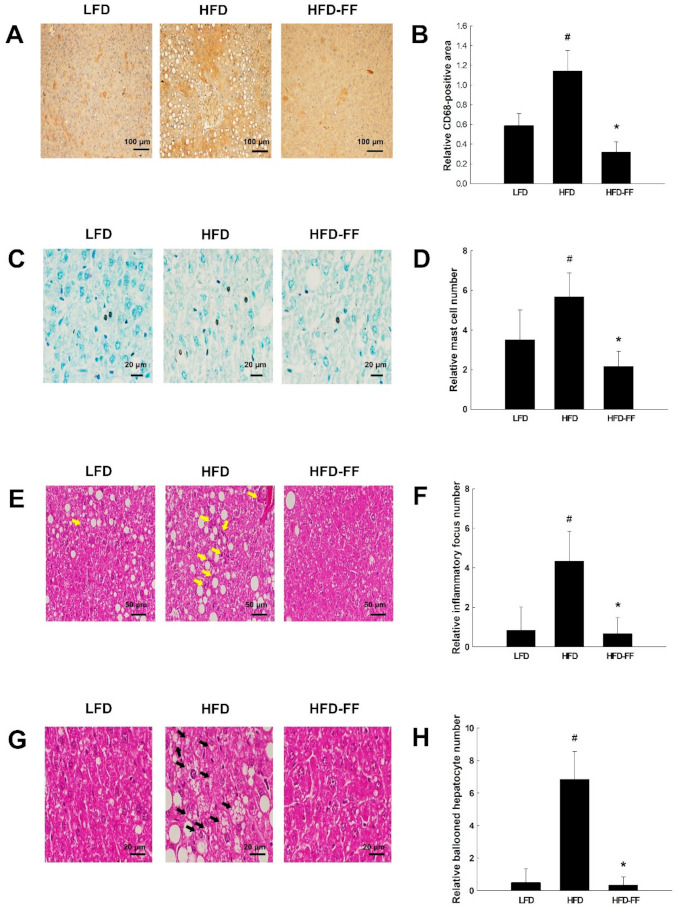
Effects of fenofibrate on hepatic inflammation and hepatocyte ballooning in HFD-fed female OVX mice. OVX C57BL/6J mice were fed an LFD, an HFD or an HFD supplemented with fenofibrate (HFD-FF, 0.05% *w*/*w*) for 21 weeks. (**A**) Immunohistochemical detection of CD68-positive macrophages in liver tissues (original magnification ×200). (**B**) Relative CD68-positive area in liver tissues. (**C**) Toluidine blue-stained sections of liver tissues (original magnification, ×400). (**D**) Relative mast cell number. (**E**) Hematoxylin-eosin-stained necroinflammatory focus of liver sections (original magnification ×100). Necroinflammatory foci are indicated by yellow arrows. (**F**) Relative inflammatory focus number. (**G**) Hematoxylin-eosin-stained ballooned hepatocytes in liver sections (original magnification ×100). Ballooned hepatocytes are indicated by black arrows. (**H**) Relative ballooned hepatocyte number. All values are expressed as mean ± SD (*n* = 8 per group). # *p* < 0.05 compared with LFD, * *p* < 0.05 compared with HFD.

Hematoxylin-eosin-stained liver sections showed that HFD feeding induced microvesicular and macrovesicular steatosis (Figure 3A); however, fenofibrate treatment reduced intrahepatic triglyceride accumulation in HFD-fed obese OVX mice (Figure 3B). We observed hepatic inflammation in HFD mice, as evidenced by liver sections stained for CD68, which is highly expressed by macrophages and monocytes; CD68-positive cells were decreased in HFD-FF mice compared with HFD mice (Figure 4A,B). Toluidine blue-stained mast cells (Figure 4C,D) and necroinflammatory foci (Figure 4E,F) were also decreased in HFD-FF mice. We also observed hepatocyte ballooning in HFD mice, as evidenced by increases in inflated cells related to hepatocyte injury (Figure 4G,H). Ballooned hepatocytes were almost completely abolished following fenofibrate treatment.

### 2.4. Effects of Fenofibrate on Visceral Adipose Expression of PPARα Target Genes in HFD-fed OVX Mice 

**Figure 5:**
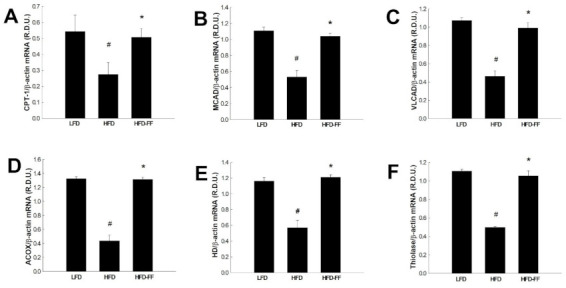
Effects of fenofibrate on visceral adipose tissue expression of genes related to fatty acid β-oxidation in HFD-fed female OVX mice. OVX C57BL/6J mice were fed an HFD or an HFD supplemented with fenofibrate (HFD-FF, 0.05% *w*/*w*) for 21 weeks. The mRNA expression of (**A**) CPT-1, (**B**) MCAD, (**C**) VLCAD, (**D**) ACOX, (**E**) HD, and (**F**) thiolase in visceral adipose tissues. All values are expressed as the mean ± SD (*n* = 8/group). # *p* < 0.05 compared with LFD, * *p* < 0.05 compared with HFD. ACOX, fatty acyl-CoA oxidase; CPT-1, carnitine palmitoyltransferase I; HD, enoyl-CoA hydratase/3-hydroxyacyl-CoA dehydrogenase; MCAD, medium-chain acyl-CoA dehydrogenase; VLCAD, very long-chain acyl-CoA dehydrogenase.

To investigate whether the inhibitory effects of fenofibrate on visceral obesity, as well as hepatic steatosis and inflammation, in obese OVX mice were caused by changes in PPARα target gene expression in visceral adipose tissue, we measured mRNA levels of PPARα target genes that encode enzymes involved with fatty acid β-oxidation, specifically carnitine palmitoyl transferase I (CPT-1), medium-chain acyl-CoA dehydrogenase (MCAD), very long-chain acyl-coenzyme A dehydrogenase (VLCAD), acyl-CoA oxidase (ACOX), enoyl-CoA hydratase/3-hydroxyacyl-CoA dehydrogenase (HD), and thiolase. HFD mice had lower expression of all the above genes compared with LFD mice (data not shown). However, in HFD-FF fed mice, fenofibrate treatment increased PPARα target gene expression to the levels of LFD mice. Fenofibrate upregulated CPT-1, MCAD, VLCAD, ACOX, HD, and thiolase mRNA levels by 240%, 176%, 188%, 128%, 143%, and 152%, respectively, relative to mice fed an HFD alone (Figure 5).

### 2.5. Effects of Fenofibrate on Visceral Adipose Inflammation in HFD-fed OVX Mice

**Figure 6:**
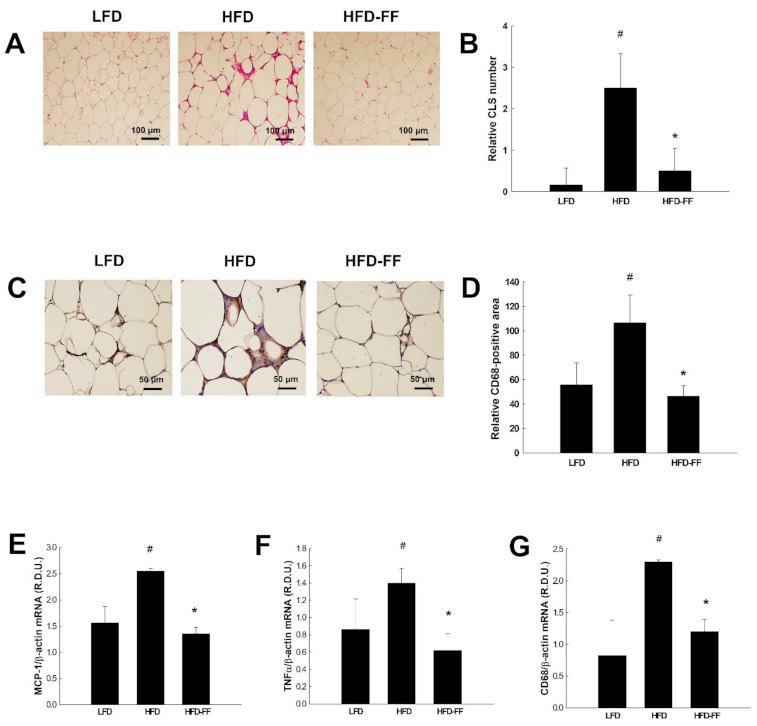
Effects of fenofibrate on visceral adipose tissue inflammation in HFD-fed female OVX mice. OVX C57BL/6J mice were fed an LFD, an HFD or an HFD supplemented with fenofibrate (HFD-FF, 0.05% *w*/*w*) for 21 weeks. (**A**) Hematoxylin and eosin-stained CLS of visceral adipose tissues (original magnification ×100). (**B**) Relative CLS number. (**C**) Immunohistochemical detection of CD68-positive macrophages in visceral adipose tissues (original magnification ×200). (**D**) Relative CD68-positive area. The mRNA expression of (**E**) MCP-1, (**F**) TNFα, and (**G**) CD68 in visceral adipose tissues. All values are expressed as mean ± SD (*n* = 8/group). # *p* < 0.05 compared with LFD, * *p* < 0.05 compared with HFD. CLS, crown-like structures; MCP-1, monocyte chemoattractant protein 1; TNFα, tumor necrosis factor α.

HFD feeding resulted in the formation of crown-like structures (CLS) in visceral adipose tissues (Figure 6A,B), with fewer being formed in HFD-FF mice. CD68-positive cells were also reduced in visceral adipose tissue (Figure 6C,D) and the expression of inflammatory genes was significantly lower, as shown by reduced mRNA levels of monocyte chemoattractant protein 1 (MCP-1), tumor necrosis factor α (TNFα), and CD68, for HFD-FF mice relative to HFD mice (Figure 6E–G).

## 3. Discussion

The incidence of obesity is higher in postmenopausal women than in age-matched pre-menopausal women, with more visceral fat accumulation as well, leading to an increased risk of the complications associated with obesity, such as dyslipidemia, type 2 diabetes, hypertension, and NAFLD [19,20]. In particular, the prevalence of NAFLD is strongly associated with visceral obesity. The association between visceral fat and liver inflammation and fibrosis is independent of the metabolic syndrome, suggesting that visceral fat is the key mediator of NAFLD [13]. We previously demonstrated that fenofibrate regulates obesity and lipid metabolism by activating hepatic PPARα actions in obese male low-density lipoprotein receptor-null and female OVX mice [8,23]. Thus, we hypothesized that fenofibrate inhibits visceral obesity and NAFLD by enhancing visceral adipose tissue PPARα action in obese female OVX mice. 

Our previous study showed that OVX mice have higher body weight, visceral fat mass, and adipocyte size following 15 weeks of HFD feeding than control non-OVX mice [22]. In the present study, fenofibrate reduced ovariectomy-induced visceral obesity and adipocyte hypertrophy. A reduction in body weight gain, correlated with a decrease in visceral fat mass, was observed 6 weeks after fenofibrate treatment in HFD mice. These results indicate that fenofibrate may decrease adiposity, which may in turn lead to a reduction in body weight. Fenofibrate also decreased the average size of the visceral adipocytes in OVX mice. Visceral obesity and adipocyte hypertrophy have been shown to increase adipokine secretion, which has been associated with metabolic diseases [18,24], suggesting that fenofibrate may be effective in preventing obesity and obesity-associated diseases in female OVX mice.

Obesity is characterized by adipose inflammation and adipocyte hypertrophy, contributing to macrophage infiltration of visceral adipose tissue [16,25]. Hypertrophied adipocytes secrete a large number of inflammatory factors, such as MCP-1 and TNFα, which stimulate macrophage infiltration and modulate the adipose tissue inflammatory response [26,27]. Macrophage levels in adipose tissue are under 10% for lean mice and humans but up to 40% for severely obese humans and mice [25]. Arrangements of macrophages in CLS around single, large adipocytes that exhibit features of necrosis have been shown in obese individuals [16,28]. In agreement with previous results that showed OVX mice have larger adipocytes relative to control non-OVX mice [22], we observed pronounced visceral adipose tissue inflammation, as indicated by CD68-positive macrophages and CLS around adipocytes in OVX mice, and fenofibrate decreased the number of infiltrating macrophages and CLS in OVX mice. The augmented expression of inflammatory genes is directly related to visceral adipose tissue dysfunction in HFD-fed obese mice [29]. In the present study, fenofibrate reduced mRNA expression of inflammatory genes, such as MCP-1, TNFα, and CD68, in the visceral adipose tissue of HFD-fed OVX mice. These results indicate that fenofibrate reduces visceral adipose tissue inflammation in obese OVX mice. The portal hypothesis proposes that free fatty acids and cytokines released from visceral adipose tissue are drained into the portal vein and have a direct connection to liver circulation. Consequently, increased production of fatty acids and inflammatory cytokines by visceral fat tissues in obese humans may have deleterious effects on liver function [15,16]. Our results suggest that fenofibrate-induced decreases in visceral adipose inflammation may lead to attenuated NAFLD. 

Visceral fat accumulation is an indicator of increased ectopic fat in other sites, including the liver, skeletal muscle, heart, and pancreas [15]. Also, increased visceral fat has been linked to obesity comorbidities and increased risk of hepatic disease. Studies from obese NAFLD subjects suggest that about 60% of hepatic triglycerides are derived from circulating free fatty acids, while 60–80% arise from adipose tissue lipolysis [30]. Mulder et al. reported that visceral adipose tissue became inflamed after HFD feeding between 12 and 24 weeks, which coincided with hepatic steatosis [17]. However, removal of inflamed visceral adipose tissue mitigated the development of NASH during obesity, suggesting that large adipocytes and adipose tissue macrophages lead to chronic inflammation with hepatic triglyceride accumulation. 

The prevalence of NAFLD in females increases with age, especially after menopause and ovariectomy-induced hepatic lipid accumulation [22,31,32]. We observed that in OVX mice, 21 weeks of HFD feeding induced liver macrovesicular and microvesicular steatosis. Liver steatosis is defined by the presence of visible intracellular triglycerides in more than 5% of hepatocytes. The severity of NAFLD is graded based upon the percentage of hepatocytes with these visible intracellular triglycerides: light NAFLD is indicated by ˂30%, moderate NAFLD 30–60%, and severe NAFLD ˃60% [33]. Our results revealed that visible triglycerides were present in more than 60% of hepatocytes in HFD-fed OVX mice, and fenofibrate inhibited hepatic triglyceride accumulation, indicating that it reduces fat accumulation in the livers of female OVX mice. These results are supported by previous reports that suggest fenofibrate improves hepatic steatosis in male NAFLD animal models, such as Otsuka Long-Evans Tokushima fatty rats, low-density lipoprotein receptor-null mice, and choline-deficient HFD-fed mice [8,34,35]; however, clinical studies have reported that fenofibrate did not significantly reduce hepatic steatosis [36,37]. In contrast to our results, Yan et al. reported that fenofibrate increased liver triglyceride accumulation in male C57BL/6J mice [38]. The discrepancy in these results may be due to differences in the animal models used for analysis. We used an animal model of obesity and NAFLD, in which mice were fed an HFD for 21 weeks. In contrast, Yan et al. used mice that were not obese, did not exhibit NAFLD, and did not receive an HFD.

Severe steatosis is more often related to inflammation, hepatocyte ballooning, and fibrosis in adult subjects with NAFLD [39]. We observed increased hepatic inflammation, inflammatory focus, and hepatocyte ballooning in HFD-fed obese OVX mice. However, fenofibrate decreased the infiltration of inflammatory cells (i.e., CD68-positive macrophages and toluidine blue-stained mast cells) into the livers of obese OVX mice. Similarly, male PPARα-KO mice have been reported to exhibit an increased abundance of macrophages in the liver compared with the liver of wild-type mice [40]. In the same study, the potent PPARα agonist Wy14,643 downregulated the expression of inflammatory genes, whereas hepatic triglyceride contents were not significantly changed. Also, we observed that fenofibrate reduced ballooned hepatocytes, defined as large swollen hepatocytes with rarified cytoplasm. Hepatocyte ballooning represents a special form of liver cell degeneration related to cell swelling and enlargement. Thus, our data suggests that fenofibrate effectively inhibits hepatic macrophage infiltration and hepatocellular ballooning in obese female OVX mice.

PPARα is mainly expressed in tissues with high rates of fatty acid catabolism, such as liver, skeletal muscle, heart, and brown adipose tissue [41,42]. In addition to the critical roles of PPARα in fatty acid oxidation in the liver and skeletal muscle, PPARα activation may regulate adipose tissue metabolism. For example, administration of bezafibrate, a conventional PPAR activator, increases fatty acid oxidation, leading to dedifferentiation of adipocytes into preadipocyte-like cells in rat primary cultures of adipocytes [43]. Likewise, the PPARα ligand GI259578A decreases the average size of adipocytes in white adipose tissue [44]. These observations are supported by our previous reports that showed that fenofibrate stimulates fatty acid β–oxidation in the visceral adipose tissue of male obese mice and differentiated 3T3-L1 adipocytes [24]. The inhibition of fatty acid oxidation has been reported to play an important role in the development of obesity and related metabolic disorders [45,46,47]. In contrast, the stimulation of fatty acid oxidation enzymes in adipocytes can exert beneficial effects on lipid accumulation-related metabolic diseases [24,48,49], suggesting that interventions targeting the fatty acid oxidation pathway can be a potential means towards preventing or treating obesity and metabolic disorders. Thus, we next evaluated whether the decreases in visceral obesity and NAFLD following fenofibrate treatment in obese OVX mice may have resulted from the stimulatory effect of fenofibrate on the adipose tissue expression of PPARα target genes, which are responsible for fatty acid β-oxidation. We observed that fenofibrate increased mRNA levels of CPT-1, MCAD, and VLCAD, which act as mitochondrial fatty acid-oxidizing enzymes in the visceral adipose tissue. Further, fenofibrate enhanced the expression of ACOX, HD, and thiolase, which oxidize fatty acids in peroxisomes. These results suggest that visceral adipose PPARα activation in obese OVX mice may lead to amelioration of visceral obesity and NAFLD.

Long-term intake of an HFD induces liver injury and increases the levels of circulating liver damage markers ALT and AST [18,50]. After 21 weeks of HFD administration, OVX mice had increased plasma ALT and AST levels compared with LFD-fed OVX mice, and fenofibrate decreased these levels. In addition, OVX mice had increased circulating levels of total cholesterol, free fatty acids, and triglycerides compared with control non-OVX female mice [22,51]. Fibrates, including fenofibrate, are a class of lipid-lowering drugs used to treat patients with hypertriglyceridemia and hypercholesterolemia [2]. Thus, as expected, fenofibrate significantly decreased plasma levels of total cholesterol, free fatty acids, and triglycerides in HFD-fed OVX mice. These results indicate that fenofibrate inhibits liver damage and dyslipidemia in obese OVX mice. 

## 4. Materials and Methods

### 4.1. Animal Treatments

Eight-week-old female wild-type C57BL/6J mice (*n* = 8/group) were purchased from Central Lab Animal (Seoul, Korea). Mice were OVX, divided into three groups (*n* = 8/group), and fed for 21 weeks with an LFD (13 kcal% fat, Research Diets, New Brunswick, NJ, USA), an HFD (45 kcal% fat, Research Diets), or an HFD supplemented with 0.05% (*w/w*) fenofibrate (HFD-FF). For the preparation of HFD-FF, 0.5 g FF was mixed with 1 kg HFD. Since a mouse consumes about 3 g of diet per day, the daily FF intake is about 1.5 mg. The body weight of each animal was measured three times per week by a person blinded to the treatments. After an 8-h fast on the last day of the study, the animals were sacrificed by cervical dislocation. Plasma levels of ALT, AST, total cholesterol, and triglycerides were measured using a blood chemical analyzer (Roche Diagnostics, Mannheim, Germany). Plasma levels of free fatty acids were measured using SICDIA NEFAZYME (Shinyang Chemical, Seoul, Korea). All animal experiments were approved by the Institutional Animal Care and Use Committees of Mokwon University and followed National Research Council Guidelines.

### 4.2. Histological Analysis

Tissue specimens were fixed in 10% phosphate-buffered formalin for 1 day and embedded in paraffin. For hepatic steatosis and inflammation analysis, liver tissue sections (5 μm) were cut and stained with hematoxylin-eosin and toluidine blue, respectively. To measure the size of adipocytes and the number of CLS, visceral adipose tissue sections were stained with hematoxylin-eosin. For quantitation of stained preparations, 5 tissue sections per animal were evaluated and 20 images from nonoverlapping fields per section were captured with a digital camera (Olympus DP70, Shinjuku, Tokyo, Japan). Images were analyzed and the numbers of CLS, mast cells, inflammatory foci, and ballooned hepatocytes were counted under microscopic observation.

### 4.3. Immunohistochemistry

Infiltrated macrophages were detected using a monoclonal mouse anti-CD68 antibody (ab955, Abcam, Cambridge, UK). Liver sections were incubated with a CD68 (1:200 dilution) primary antibody, followed by an anti-mouse IgG biotinylated secondary antibody (Vector Laboratories, Burlingame, CA, USA) with diaminobenzidine (Vector Laboratories) as a color substrate. Visceral adipose tissue sections were immunostained with an anti-CD68 antibody and counterstained with Mayer’s hematoxylin. For quantitation of immunostained CD68-positive area, 5 sections of liver or visceral adipose tissue per animal were evaluated and 20 images per section were analyzed using ImageJ software (http://imagej.nih.gov/ij/, accessed on 24 March 2021). Relative immunostained areas were expressed as a percentage of the total surveyed areas.

### 4.4. Determination of Hepatic Triglyceride Levels

Hepatic triglyceride levels were measured using an ab65336 Triglyceride Quantification Assay Kit (Abcam). Briefly, total lipids from liver tissues were extracted with distilled water containing 5% Nonidet P-40, heated at 80–100 °C for 2–5 min, and cooled down to room temperature. After homogenates were centrifuged, supernatants were mixed with lipase, triglyceride assay buffer, and triglyceride reaction mix. After 60 min, optical density was estimated on a microplate reader at 570 nm.

### 4.5. Quantitative Reverse Transcription Polymerase Chain Reaction (qRT-PCR)

Total RNA from visceral adipose tissues was prepared using Trizol reagent (Invitrogen, Carlsbad, CA, USA). Complementary DNA (cDNA) was synthesized from 2 μg total RNA using the TOPscript^TM^ DryMIX RT kit containing reverse transcriptase, RT buffer, a deoxyribonucleotide triphosphate mixture, RNase inhibitor, and oligo (dT)18 (Enzynomics, Seoul, Korea) in a final volume of 20 μL according to the manufacturer’s instructions. Briefly, RT reactions were incubated for 60 min at 50 °C and then inactivated by heating to 95 °C for 10 min. The genes of interest were amplified from the synthesized cDNA using BioFact ^TM^ 2X Real-Time PCR Master Mix (BioFact, Daejeon, Korea) and a Rotor-Gene 6000 system (Qiagen, Hilden, Germany). The PCR primers used for gene expression analysis are shown in Table S1 (Supplementary data). PCR was performed using the following conditions: 1 cycle of 95 °C for 15 min, followed by 50 cycles of 95 °C for 20 s, 58 °C for 15 s, and 72 °C for 20 s. The relative expression levels were calculated as the ratio of the target gene cDNA to 18S cDNA. 

### 4.6. Statistical Analysis 

Values were expressed as mean ± standard deviation (SD). Statistical analysis was performed using analysis of variance followed by Turkey’s post-hoc tests. Statistical significance was defined as *p* < 0.05. 

## 5. Conclusions

In conclusion, the results of this study provide evidence that fenofibrate can regulate visceral obesity and NAFLD, partly through visceral adipose tissue PPARα activation, in obese OVX mice. In addition, fenofibrate may protect the livers of obese OVX mice against hypertriglyceridemia and hypercholesterolemia. Our results suggest that fenofibrate may be effective in regulating obesity and NAFLD in estrogen-deficient obese states.

## Data Availability

The data that support the findings of this study are available from the corresponding author upon reasonable request.

## Supplementary Materials

The following are available online at https://www.mdpi.com/article/10.3390/ijms22073675/s1, Table S1: Sequences of primers used for quantitative real-time PCR assays.
