# Supplementary material for: Fenofibrate Regulates Visceral Obesity and Nonalcoholic Steatohepatitis in Obese Female Ovariectomized C57BL/6J Mice
Source: Int J Mol Sci. 2021 Apr 1;22(7):3675. doi: 10.3390/ijms22073675 (PMC8038108; doi:10.3390/ijms22073675)
Supplement: Supplementary file 1 [file ijms-22-03675-s001.pdf]

Table S1. Sequences of primers used for quantitative real-time PCR assays

Genes	Gene Bank No.	Primer sequence	
ACOX	NM_015729.3	Forward :	5`-GCCCAACTGTGACTTCCATT-3`
		Reverse :	5`-GGCATGTAACCCGTAGCACT-3`
CD68	NM_001291058.1	Forward :	5`-CCCACAGGCAGCACAGTGGAC-3`
		Reverse :	5`-TCCACAGCAGAAGCTTTGGCCC-3`
CPT-1	NM_013495.2	Forward :	5`-CAGCAGCAGGTGGAACTGT-3`
		Reverse :	5`-GGAAACACCATAGCCGTCAT-3`
HD	NM_023737.3	Forward :	5`-ACTTGCATGGGTATGGGTGG-3`
		Reverse :	5`-GCTGGGGGATGTCAGGATTC-3`
MCAD	NM_007382.5	Forward :	5`-TGATCAACGCGCACATTC-3`
		Reverse :	5`-GAACGTTCCCAGGCCAAG-3`
MCP-1	NM_011333.3	Forward :	5`-CCCCAAGAAGGAATGGGTCC-3`
		Reverse :	5`-GTGCTGAAGACCTTAGGGCA-3`
Thiolase	NM_009230.3	Forward :	5`-TACCCAAGGACTCCTACTGTAAG-3`
		Reverse :	5`-ACCAGGACACGAGCACTGA-3`
TNFα	NM_013693.3	Forward :	5`-CAGGCGGTGCCTATGTCTC-3`
		Reverse :	5`-CAGTCACCCCGAAGTTCAGTAG-3`
VLCAD	NM_017366.3	Forward :	5`-GCCCAGACACACAACCTTTG-3`
		Reverse :	5`-CCGAGCCGACTGCATCTC-3`
18S	NR_003278.3	Forward :	5`-GCAATTATTCCCCATGAAC-3`
		Reverse :	5`-GGCCTCACTAAACCATCCAA-3`
